# Undesirable Status of Prostate Cancer Cells after Intensive Inhibition of AR Signaling: Post-AR Era of CRPC Treatment

**DOI:** 10.3390/biomedicines9040414

**Published:** 2021-04-12

**Authors:** Tomoyuki Makino, Kouji Izumi, Atsushi Mizokami

**Affiliations:** Department of Integrative Cancer Therapy and Urology, Kanazawa University Graduate School of Medical Science, Kanazawa 920-8641, Japan; mackeeen511@gmail.com (T.M.); mizokami@staff.kanazawa-u.ac.jp (A.M.)

**Keywords:** androgen receptor, castration-resistant prostate cancer, neuroendocrine prostate cancer, double-negative castration-resistant prostate cancer

## Abstract

Recent advances in prostate cancer (PC) research unveiled real androgen receptor (AR) functions in castration-resistant PC (CRPC). Moreover, AR still accelerates PC cell proliferation via the activation of several mechanisms (e.g., mutation, variants, and amplifications in CRPC). New-generation AR signaling-targeted agents, inhibiting extremely the activity of AR, were developed based on these incontrovertible mechanisms of AR-induced CRPC progression. However, long-term administration of AR signaling-targeted agents subsequently induces the major problem that AR (complete)-independent CRPC cells present neither AR nor prostate-specific antigen, including neuroendocrine differentiation as a subtype of AR-independent CRPC. Moreover, there are few treatments effective for AR-independent CRPC with solid evidence. This study focuses on the transformation mechanisms of AR-independent from AR-dependent CRPC cells and potential treatment strategy for AR-independent CRPC and discusses them based on a review of basic and clinical literature.

## 1. Introduction

Androgen receptor (AR) signaling is a hallmark of prostate cancer (PC). Moreover, androgen deprivation therapy (ADT) is highly effective in most patients with metastatic PC. However, resistance eventually develops, leading to a lethal phenotype known as metastatic castration-resistant PC (mCRPC) [1]. An important CRPC feature is the reactivation of AR signaling, represented by progressive rises in serum prostate-specific antigen (PSA), which is a gene product transcriptionally regulated by AR [1]. Several mechanisms have been established to maintain AR activity (e.g., AR mutation, amplification, and splice variants) [2,3,4]. Therefore, the role of AR signaling in CRPC is still very important and therapeutics directed toward further suppressing AR ligands or AR itself (AR signaling axis) have been developed.

Although neuroendocrine (NE) PC (NEPC) rarely arises de novo and accounts for <2% of patients at the time of PC diagnosis [5], treatment-emergent NEPC (t-NEPC) has been recently recognized to be observed in later stages of PC progression in up to 15–20% of patients treated with AR signaling-targeted agents (ARSTAs), including abiraterone acetate and enzalutamide, in the metastatic and castration-resistant disease state [6,7,8]. This transdifferentiation is a process, also known as lineage plasticity, by which a differentiated cell type transitions to another lineage to evade the pressure of therapeutically relevant drugs [9,10]. PC cells try to escape the inhibition of the AR pathway and transform from AR-driven adenocarcinoma to t-NEPC. This study also recently revealed that ARSTAs potentially promote NE differentiation and visceral metastases, and the prognosis of CRPC patients who developed new visceral metastases after treatment was extremely poor [11]. Although PC relapsing from ADT may exhibit mutant histologies with altered expression of lineage markers, suggesting that lineage plasticity promotes treatment resistance, the mechanisms underlying phylogenetic plasticity in PC are still poorly understood. To focus on the phenotypic and molecular landscapes of CRPC, the present article aims to classify complex spectrum of phenotypes into three subsets of CRPC, AR-dependent CRPC, and AR-independent CRPC including NEPC and PSA-null and NE-null CRPC (double-negative CRPC; DNPC), and on the mechanisms of transformation of AR-independent CRPC cells from AR-dependent CRPC cells and potential treatment strategy for AR-independent CRPC. Moreover, they are discussed based on a review of basic and clinical literature.

## 2. The Role of Androgen Receptor (AR) in AR-Dependent Castration-Resistant Prostate Cancer (CRPC)

Several therapies have been developed and approved over the last decade, suggesting the importance of sequencing systemic therapy in mCRPC. The prognosis for mCRPC is generally reported to be 15–32 months depending on the nature of the clinical trial [12]. Most mCRPC patients eventually develop lethal disease despite dramatic therapeutic advances, not to mention that AR signaling is still involved in the background.

### 2.1. Secondary AR Alterations

#### 2.1.1. AR Mutation

Point mutations in the AR gene were found in 10–30% of CRPC patients [13,14]. The majority of AR mutations identified in PC samples are point mutations resulting in a single amino acid substitution [15]. While these mutations are predominantly localized to the ligand-binding domain (LBD) [16], mutations in the amino terminal and DNA-binding domains were also identified [14]. For example, mutations in the LBD of AR (H874Y, T877A, and T877S) have been shown to relieve the AR ligand specificity and allow activation by adrenal androgens or alternative steroid hormones (e.g., cortisol or progesterone) [17]. Other mutations, including T878A and F876L, show agonistic effects with treatment with AR antagonists flutamide and bicalutamide, respectively, as well as with new-generation AR antagonists such as enzalutamide [18,19]. Prominent AR mutations and their effects on AR activity are summarized well in another review [20].

#### 2.1.2. AR Amplification

AR gene amplification was found in 20–30% of CRPC patients, which is uncommon in hormone-naïve PC, resulting in AR overexpression [21,22]. An elevated level of AR gene expression could contribute to the hypersensitivity of the low level of androgen state under castration status, which promotes disease progression [23,24]. Importantly, recent studies have identified a strong amplified upstream AR enhancer, which may be a key driver for increased AR expression [25,26,27]. As other possible players in this mechanism, deregulation of transcription factors and/or coregulators could be involved. The nuclear factor kappa B (NF-κB) has shown to bind directly to AR promoter and enhancer and increase the levels of both AR mRNA and AR proteins [28].

#### 2.1.3. AR Splice Variants

More recent studies have identified splice variants of ARs (AR-Vs) that are constitutively activated by the loss of the C-terminal LBD [29,30,31]. AR-V1 and AR-V7 were the most abundant variants with a 20-fold higher expression in CRPC compared with hormone-naïve PC [32]. Furthermore, AR-V7 is located in the nuclei and constitutively activated under androgen-deprived conditions. ARSTAs (e.g., abiraterone acetate and enzalutamide) could induce AR-V7 expression in circulating tumor cells, whereas chemotherapy (e.g., docetaxel and cabazitaxel) diminished the AR-V7 expression [33,34]. These AR-V7 changes in circulating tumor cells may reflect the selective pressures on PC cells using the treatments. Similar to AR-V7, ARv567es is constitutively active and translatable into protein despite the lack of specific antibodies. ARv567es have been proposed as another AR variant-dependent resistance mechanism because several studies have confirmed the ARv567es expression in PC specimens [35,36,37,38]. In contrast, AR-V7 is also useful as a prognostic marker due to its overexpression that is associated with an increased risk of biochemical recurrence after radical prostatectomy in hormone-naïve PC patients and shorter survival in CRPC [37,39]. AR splice variants known to lack the LBD are well summarized in another review [40].

### 2.2. AR Bypass/Crosstalk Mechanisms

#### 2.2.1. Growth Factors

Growth factors such as insulin-like growth factor-1 (IGF-1), keratinocyte growth factor, and epidermal growth factor (EGF) activate AR and allow the induction of transactivation of AR target genes under low androgen conditions [41]. IGF-1 has been the most extensively studied of the growth factors and shown to enhance AR signaling even in the absence of androgens. IGF-1 has also been shown to promote AR signaling by increasing the expression of various AR coactivators (e.g., transcriptional intermediary factor 2 and insulin-degrading enzymes) [42,43]. Similarly, EGF signaling activates AR in a ligand-independent manner and induces transcription of AR-regulated genes [41]. These growth factors are ligands for receptor tyrosine kinases (RTKs), indicating the importance of RTK signaling in PC progression. These receptors are known to induce downstream activation of essential growth and survival pathways, including AKT, mitogen-activated protein kinase (MAPK), and signal transducer and activator of transcription (STAT) pathways [44].

#### 2.2.2. Cytokines

Along with growth factors, a variety of cytokines activate AR as well. The NF-κB signaling pathway, which is constitutively expressed in many different types of cancer and involved in tumor formation and progression [45], regulates the cytokines interleukin-6 (IL-6) and interleukin-8 (IL-8). Increased NF-κB signaling leads to AR activation in PC cells, and AR activation is inhibited by blocking the NF-κB signaling pathway. Similarly, IL-6 and IL-8 are also known to stimulate AR activity and enhance the expression of AR-regulated genes [46,47,48].

#### 2.2.3. Phosphatidylinositol-3 Kinase (PI3K)/AKT Pathway

The phosphatidylinositol-3 kinase (PI3K)/AKT pathway is an important player in the CRPC deterioration process. The loss of the tumor suppressor phosphatase and tensin homolog deleted on chromosome 10 (PTEN) protein, a negative inhibitor of the PI3K/AKT pathway, has been identified in almost all metastatic PC. Its activation has been associated with the CPRC development in a variety of preclinical models [49,50]. Microtranscriptomic PC analysis was recently performed to identify the crosstalk pathway between AR and PI3K/AKT signaling [51]. The result revealed reverse feedback in which a loss of negative feedback is identified under conditions of either androgen deprivation or PI3K inhibition, resulting in the reverse pathway activation. Thus, the inhibition of one oncogenic pathway activates the other, providing another potential resistance mechanism to AR inhibition [52,53]. Therefore, the combined PI3K/AKT inhibition and AR signaling results in almost complete tumor regression, prolonged tumor growth inhibition, and PSA stabilization in a preclinical CRPC model [53].

#### 2.2.4. Wnt Pathway

The canonical Wnt pathway is also thought to be associated with the CRPC deterioration process. Moreover, β-catenin, which is a major downstream effector of the Wnt pathway, acts as an important AR coactivator, augmenting AR-mediated transcription [54]. More interestingly, the activation of the Wnt/β-catenin pathway led to drug resistance in enzalutamide-sensitive cells, and the combination of the β-catenin inhibitor and enzalutamide synergistically suppressed stem-like marker expression, cell proliferation, and tumor growth in various models [55]. Therefore, crosstalk between the Wnt/β-catenin and AR pathway is another important interaction that promotes CRPC.

#### 2.2.5. Glucocorticoid Receptor Upregulation

**Figure 1:**
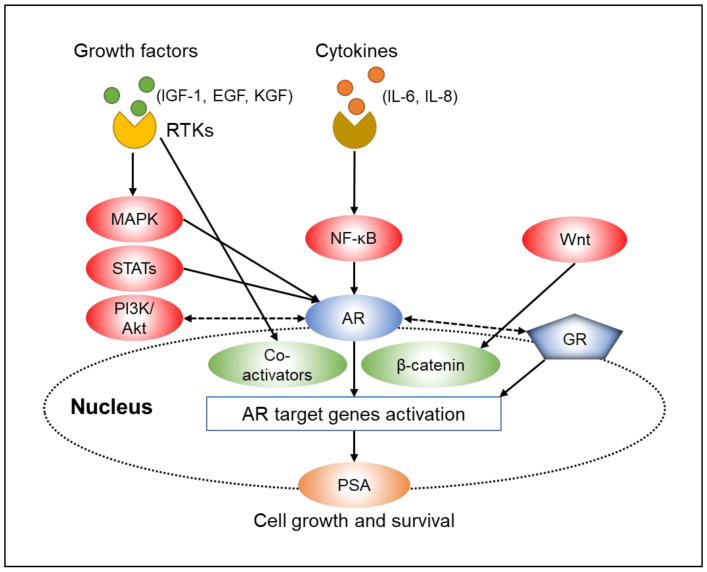
Schematic presentation of the androgen receptor (AR) bypass/crosstalk mechanisms. AR androgen receptor, EGF epidermal growth factor, GR glucocorticoid receptor, IGF-1 insulin-like growth factor-1, IL interleukin, KGF keratinocyte growth factor, PSA prostate-specific antigen, RTK receptor tyrosine kinases. Solid arrows indicate activation and broken arrows indicate interaction.

Acquired resistance to abiraterone acetate and enzalutamide may occur via increased expression of the glucocorticoid receptor (GR), which share response elements with AR in multiple gene targets [56]. In addition, increased GR expression and activity have been shown to contribute to tumor-promoting PC cell survival [57]. AR and GR are mutually balanced in their expression. AR inhibition causes an increase of GR levels due to the loss of AR-mediated negative feedback [58]. Therefore, targeting the GR pathway in combination with antiandrogens may be a promising treatment strategy for advanced PC [59]. Schematic presentation of the AR bypass/crosstalk mechanisms is shown in Figure 1.

## 3. Neuroendocrine Prostate Cancer (NEPC)

### 3.1. Definition

In 2013, the Prostate Cancer Foundation assembled a working committee on the molecular biology and pathologic classification of NE differentiation in PC and proposed a new pathological classification of NEPC [60]. These subtypes include (a) usual prostate adenocarcinoma with NE differentiation, (b) adenocarcinoma with Paneth cell NE differentiation, (c) carcinoid tumor, (d) small-cell carcinoma, (e) large cell NE carcinoma, and (f) mixed NE carcinoma-acinar adenocarcinoma. In contrast, the aggressive-variant PC, formally termed as anaplastic PC, is clinically defined as CRPC with at least one of the following: (1) histologic evidence of small-cell PC (pure or mixed); (2) presence of exclusively visceral metastases; (3) radiographically predominant lytic bone metastases; (4) bulky (≥5 cm) lymphadenopathy or bulky (≥5 cm) high-grade (Gleason score ≥8) tumor mass in prostate/pelvis; (5) low PSA (≤10 ng/mL) at initial presentation (prior to ADT or at symptomatic progression in the castrate setting) plus high volume (≥20) bone metastases; (6) presence of NE markers on histology (positive staining of chromogranin A or synaptophysin) or in serum (abnormal high serum levels for chromogranin A or gastrin-releasing peptide) at initial diagnosis or at progression; combined with either elevated lactate dehydrogenase (LDH), malignant hypercalcemia, or elevated serum carcinoembryonic antigen (CEA); and (7) progression to CRPC in ≤6 months after initiation of hormonal therapy [61]. Moreover, PC cells with NE differentiation do not express AR [62]. However, AR-positive NE cell populations in PC have also been described [63]. Clinically, increased serum NE marker level with very low PSA level or histologically proven NE cells with immunohistochemistry may be an uncomplicated diagnostic criterion for NEPC.

### 3.2. Mechanisms

More potent ADT regimens targeting AR itself and androgen biosynthetic pathways have recently led to the emergence of resistance mechanisms independent of AR activity. This lethal mCRPC appears to adapt to ADT via lineage plasticity, rather than as a result of the emergence of resistance mutations, and adopt a phenotype that is no longer dependent on AR signaling and its expression. These tumors have NE features, a stem or basal cell-like phenotype, altered kinase signaling, and epigenetic modification [64,65]. The t-NEPC is a highly aggressive, histologically distinct PC subtype composed of small-cell carcinoma cells or mixed histology cells that is thought to emerge in response to the selective treatment pressure with potent ADT in the mCRPC state [66]. Targeting epigenetic mechanisms may be promising to reverse or delay NE alterations as the understanding of NEPC epigenome also advances [67].

Molecular features in NEPC include reduced or absent AR signaling; retinoblastoma 1 (RB1) loss; tumor protein p53 (TP53) loss; N-Myc amplification; Aurora Kinase A (AURKA) gain; upregulation of BRN2, sex determining region Y-box 2 (SOX2), or paternally expressed imprinted gene 10 (PEG10); downregulation of RE1-silencing transcription factor (REST); altered DNA methylation; and increased polycomb-mediated gene silencing through enhancer of zeste homolog 2 (EZH2) [8,68,69,70,71]. Activation of oncogenic drivers, in combination with significant epigenetic changes (e.g., EZH2 overexpression and DNA methylation), further promotes tumor growth and the expression of downstream neuronal and NE pathways [72]. N-Myc is a key driver of NEPC phenotype, and a mechanistic link between N-Myc and EZH2 has also been elucidated, in which they cooperate to drive histone methylation, repress AR signaling, and drive NEPC phenotype [71]. The combination of RB1 and TP53 deficient has also been shown to promote the development of NEPC in PC preclinical models [73,74], suggesting that RB1 and TP53 contribute to NEPC pathogenesis. The REST, a transcriptional repressor of neuronal signaling, is significantly downregulated in NEPC via the splicing regulator SRRM4, which is a potent inducer of NE differentiation under inhibition of the AR pathway and accentuated by loss of RB1 and p53 [75,76]. The neural transcription factor BRN2 has recently been shown as a major driver of NEPC phenotype and is significantly overexpressed in NEPC. Mechanistic studies have shown that BRN2 is directly repressed by AR and that this repression is lost under the selective pressure of enzalutamide, thereby promoting the development of NEPC both in vitro and in vivo [77]. Moreover, SOX2 is an important transcription factor involved in NEPC [78] and BRN2 has been reported to be an upstream regulator of SOX2 in driving NEPC [77]. Interestingly, the function of RB1 and TP53 loss has been reported to promote the activation of pluripotency network mediated, in part, through the non-repression of SOX2 as well as EZH2 [66,79,80]. This suggests that a variety of mechanisms are required to cooperate with genomic changes to direct cell fate to the NE lineage.

**Figure 2:**
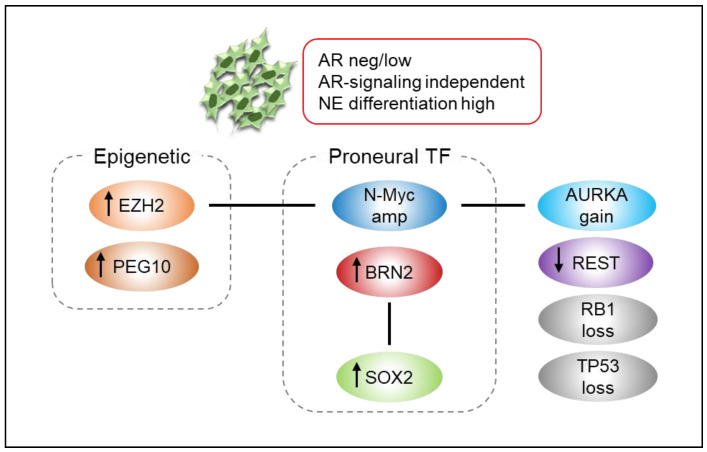
Neuroendocrine prostate cancer (NEPC) is characterized by loss of tumor suppressors, activation of oncogenic drivers, and epigenetic changes. AR androgen receptor, NE neuroendocrine, TF transcription factor. Up and down arrows indicate upregulation and downregulation, respectively.

In contrast, placental gene PEG10 is highly expressed in NEPC and is dynamically regulated by AR and E2F/RB pathways during NEPC development [81,82]. Thus, the AR antagonist activity increases the expression of PEG10 and terminal NE markers, supporting that the stimulation of AR activity by androgen supplementation reverses the expression of PEG10 and terminal NE markers [83]. Although AURKA is a mitotic kinase that plays an important role in the early stages of mitosis by regulating the centrosome function and spindle assembly, thereby promoting cell cycle and proliferation [84]. Moreover, the upregulation of this oncogene was significantly overexpressed in NEPC [69]. Notably, AURKA interacts with N-Myc and regulates the stability of N-Myc. Their amplification is precursors of tumors at risk of promoting t-NEPC after ADT [71,85]. Moreover, a genetically engineered mouse model, in which PTEN and TP53 were inactivated, showed no response to abiraterone acetate and accelerated progression to tumors showing a variety of histologic subtypes, including not only NE differentiation but also squamous, sarcomatoid, and other non-adenocarcinoma phenotypes [86]. Schematic presentation of the NEPC-related molecules and their roles is shown in Figure 2.

### 3.3. Diagnosis

#### 3.3.1. Serum Markers

The use of NE serum markers appears to be more suitable to diagnose NEPC as the majority of NE cells store neuropeptides in cytoplasmic granules, including chromogranin A (CgA) and neuron-specific enolase (NSE). The serum levels of these neuropeptides are associated with the degree of NE differentiation in PC cells [87].

##### Chromogranin A (CgA)

Published data report a correlation between the number of NE cells in the prostate-expressing CgA and serum CgA levels. An initial increase in serum CgA may indicate a disease that is only weakly sensitive to androgen deprivation. Furthermore, serum levels of CgA can potentially increase at the moment of metastatic spread. Therefore, CgA can be used as a tumor volume marker [88].

##### Neuron-Specific Enolase (NSE)

Although serum levels of NSE do not correlate directly with marker expression in pathological specimens, it is significantly higher in PC with metastatic cases than in localized forms [89]. In contrast, the elevated levels of NSE and/or CgA were associated with shorter PSA progression-free survival (PFS), clinical or radiographic PFS, and overall survival (OS) in patients with mCRPC treated with abiraterone acetate. This may be due to an increased risk of rapid resistance development with abiraterone acetate, associated with NE differentiation [90].

##### Progastrin-Releasing Peptide (Pro-GRP)

Pro-GRP has been identified as a biomarker for small-cell lung cancer (SCLC), an NE tissue differentiation disorder, and has been suggested to be useful in combination with NSE in monitoring the treatment of established SCLC [91]. In contrast, pro-GRP was reported to be a relatively sensitive marker in patients with small-cell NE carcinoma (the sensitivity in the lung was 76% compared with 54% in the no-lung tumors) [92]. In addition to CgA, pro-GRP is a complementary tumor marker for prognostic and therapeutic monitoring of patients with NE tumors (NETs) [93].

#### 3.3.2. Radiological Characteristics

Fluorodeoxyglucose-positron emission tomography/computed tomography (FDG-PET/CT) is useful for SCLC staging because the detection rate of primary and metastatic lesions is very high due to the strong FDG accumulation [94]. Identifying NEPC, specifically by PET/CT, is difficult although PET/CT is also useful in detecting metastases in NEPC [95]. Somastostatins (SSTs) induce their biological effects by interacting with specific receptors belonging to the superfamily. Five receptor subtypes (SSTR1–SSTR5) of G-protein-coupled receptors have been identified, all of which are expressed in normal human tissues. However, the predominant subtypes in endocrine tissues are SSTR2 and SSTR5 [96,97]. Furthermore, NETs have been shown to express SSTR2 and SSTR5 [98,99]. Given the presence of the NE element, the use of a 111In-pentetreotide scintigraphy (Octreoscan^®^) and somatostatin receptor scintigraphy (SRS), a radiolabeled somatostatin analog with high binding affinity to the SSTR, can be useful to evaluate disease extension [100,101] and also contribute to theranostic strategy, which is a new medical field that combines and integrates specific targeted treatments as well as specific targeted diagnostic tests [102,103]. Interestingly, the accumulation of FDG and SRS has been reported to be inversely correlated. Thus, the SRS positivity is higher in well-differentiated NETs with low proliferative potential, and the positivity of FDG-PET is higher than SRS in more undifferentiated NE carcinomas with a high proliferative potential [104]. Moreover, reports on the prognostic value of metastatic NETs suggest that SRS positivity and FDG-PET negativity are good prognostic factors, while SRS negativity and FDG-PET positivity are poor prognostic factors [105].

#### 3.3.3. Histological Characteristics

NEPC is diagnosed by morphological confirmation of small-cell carcinoma by hematoxylin-eosin staining and expression of NE markers by immunostaining. However, no clear diagnostic method or definition exists. In contrast, an autopsy report of 31 men who expired due to CRPC showed that a selection of NE markers was expressed in 78% of the metastatic sites [106]. Typically, the induction of CgA and synaptophysin expression, loss of AR and PSA expression, and upregulation of CD56 can help distinguish NEPC from prostatic adenocarcinoma [107]. Another well-known immunohistochemical parameter of NETs and NE differentiation is the overexpression of SSTRs. Other NE markers have been reported in the literature, although they are not commonly used in clinical practice. These markers include cytochrome b561 [108], synaptic vesicle protein 2 [109,110], vesicular monoamine transporter [111], soluble N-ethylmaleimide-sensitive factor attachment protein (SNAP) receptor (SNARE) complex (synaptobrevin, syntaxin and SNAP-25) [112], Rab3 [113], vesicle-associated membrane proteins [114,115], and several specific lymph follicular antigens expressed by NE cell (e.g., CD57) [116,117].

### 3.4. Treatment

Although NEPC currently lacks an effective treatment, platinum-containing combination chemotherapies (e.g., cisplatin/etoposide, carboplatin/etoposide, cisplatin/docetaxel, and carboplatin/docetaxel) following SCLC are mainly being employed for NEPC based on The National Comprehensive Cancer Network (NCCN) [118]. In a phase-II trial of 120 aggressive/anaplastic mCRPC patients, carboplatin/docetaxel followed by second-line cisplatin/etoposide revealed 47% PSA response (i.e., PSA decline ≥ 50%), 34% objective response (OR) of measurable disease, 5.1 months median PFS, and 16 months median OS [61]. Furthermore, in a phase-II GETUG P01 for 60 mCRPC patients with visceral metastases or elevated NE markers, carboplatin/etoposide revealed 8% PSA response, 9% OR of measurable disease, 2.9 months median PFS, and 9.6 months median OS [119]. Other regimens include cisplatin/irinotecan, carboplatin//irinotecan, gemcitabine/docetaxel/carboplatin, doxorubicin/cisplatin/etoposide, amrubicin, and everolimus [120,121,122,123,124]. Summarizing the data, the survival time is generally reported to be 7–16 months, indicating that the NEPC prognosis and aggressive/anaplastic mCRPC are very poor [11,125,126]. Although a few scattered reports were noted showing the efficacy of these treatments, none have been proven by clinical trials with a high level of evidence. Thus, further validation is needed in the future.

Moreover, a rationale for immune checkpoint inhibitors (ICIs) therapy may exist based on SCLC data. However, studies focused on NEPC have not yet been reported. A recent phase-I trial of nivolumab plus ipilimumab for advanced rare genitourinary tumors, including small cell/NEPC, is ongoing (NCT03333616). In addition to existing treatments, some of the molecules identified as being involved in NEPC progression can be potentially targeted. Currently, alisertib, a drug that inhibits the interaction between N-Myc and AURKA, showed exceptional responses in a subset of patients with NEPC in a phase-II trial [127]. Moreover, EZH2 is one of the important targets, and inhibition in preclinical models has shown promising effects [71]. Two clinical trials (NCT03460977 and NCT03480646) comparing EZH2 inhibitors alone or in combination with abiraterone acetate, enzalutamide, and prednisone in patients with CRPC are underway. Moreover, an analysis of these trials may provide additional insights. Another molecule, PEG10, is highly expressed in patients with NEPC and may be a promising therapeutic target for NEPC [83]. Nonetheless, the development of epigenetic therapies targeting NEPC remains challenging. One reason is that most epigenetic therapies have a non-specific distribution and exert their functions across a broad transcriptional network, making it impossible to achieve cancer cell specificity, leading to undesirable off-targets and toxic side effects [128]. Thus, further identifying the molecular targets as described above to improve NEPC treatment and prognosis is essential.

Recent data showed that Trop2 is a driver of metastatic PC with NE phenotype via poly-ADP ribose polymerase 1 (PARP1). PARP1 inhibition in Trop2-driven NEPC significantly reduces NE function, tumor growth, and metastatic colonization in vivo, suggesting that PARP1 inhibitors may be a promising therapeutic strategy for metastatic PC expressing high levels of Trop2 [129].

## 4. Prostate-Specific Antigen (PSA)-Null and NE-Null Double-Negative CRPC (DNPC)

### 4.1. DNPC Position

Importantly, some mCRPC did not express AR markers or NE differentiation [130]. Phenotypic changes were found to occur in mCRPC with the appearance of PSA-null and NE-null phenotypes, DNPC. Although DNPC could show a wide range of AR expression levels occasionally, its signal intensity is thought to be lost almost completely resulting in PSA-null status. Moreover, DNPC does not have any longer systemic anticancer treatment although NEPC can be narrowly treated with several agents as described and is, therefore, lethal CRPC. Interestingly, in the era before the approval of ARSTAs, including abiraterone acetate and enzalutamide, most CRPC were AR-dependent PC (88.4%) with rare NEPC (6.3%) and DNPC (5.4%), although tumor phenotypic changes with a higher percentage of DNPC (23.3%) were observed in the contemporary era [1]. Thus, potent suppression of AR signaling promotes the development of a variety of castration-resistant tumor subtypes, ranging from tumors that remain positive for AR-dependent PC and NEPC to DNPC [131].

### 4.2. Potential Mechanisms

DNPC may have overlapping epigenomic changes with NEPC. Lineage plasticity in DNPC, for example, may be driven by epigenetic reprogramming mediated by polycomb repressive complex 1 (PRC1). Moreover, PRC1 activity in DNPC leads to the upregulation of CCL2, resulting in bone and visceral metastases, stem cell properties, and the formation of an immunosuppressive tumor microenvironment [132]. Therefore, the pharmacological PRC1 inhibition may reverse these processes and work in concert with immune checkpoint blockade to suppress multiorgan site metastases. Several C–C motif ligand (CCL)-receptor (CCR) axes have been implicated in PC cell migration associated with the blockade of androgen/AR signaling [133]. Prostatic epithelial AR silencing promotes STAT3 activation and epithelial–mesenchymal transition (EMT) in PC cells via the CCL2—CCR2 axis, which may be associated with the secretory phenotype and proinvasive properties of PC cells [134]. The EMT phenotypes are frequently associated with CRPC, including DNPC, and androgen deprivation can induce EMT [135]. Thus, AR inhibition may induce EMT as a form of lineage plasticity, similar to its role in promoting NE differentiation in NEPC. Furthermore, the activation of PRC1 in DNPCs may underlie the ability of AR downregulation to stimulate lineage plasticity. Moreover, EZH2 is overexpressed in patients who progress to AR-independent CRPC. EZH2 recruits DNA methyltransferase to repress gene expression, highlighting the possibility of EZH2 as a master regulator of epigenome rearrangement [136]. The suppression of EZH2 function showed not only anticancer activity but also reverse lineage plasticity programs as well as reactivation of AR expression and sensitizing tumors to ADT [74].

DNPC was reported to have elevated fibroblast growth factor (FGF) signaling in the autocrine and paracrine manner, contributing to the avoidance of AR dependence. Moreover, FGF/MAPK pathway was activated in this DNPC [1]. FGF ligands and receptors have previously been shown to influence the development and progression of PC [137]. Consequently, MAPK signaling has also been shown to promote poorly differentiated tumor growth in PC models [138]. Constitutive ERK1/2 activity has been associated with castration resistance as well [139]. Unfortunately, the mechanisms affecting FGF expression in DNPC are unknown to date.

Other mechanisms include that overexpression of C-Myc and PTEN loss synergistically induce genomic instability and enable lethal metastatic PC development without not only NE features but also sarcomatoid differentiation [140]. Furthermore, lysine-specific demethylase 1 (LSD1) has been shown to promote the survival of CRPC cells in an AR-independent manner although LSD1, a regulator of gene expression in stem cells and cancer, is highly expressed in the tumors of patients with lethal CRPC [139]. LSD1 suppression reduces the survival of PC cells that are grown without androgens that are resistant to the AR antagonist enzalutamide or that do not even express AR. Moreover, the inhibition of LSD1 expression resulted in a greater anticancer effect when combined with ADT in vitro, suggesting that targeting LSD1 may enhance ADT or reverse resistance to ADT [141].

### 4.3. Potential Treatment

Unfortunately, no standard treatments for DNPC exist because of the loss of potential targets such as AR signaling and NE lineage. From the above background, FGF/MAPK blockade suppressed the growth of DNPC in vitro and in vivo, suggesting that FGF/MAPK inhibition may be particularly effective against mCRPC with a PSA-null phenotype [1]. The treatment of mice with GW-516, an inhibitor of PRC1, suppressed metastases of PC cells with androgen-independent or DNPC features and reduced CCL2 secretion. Furthermore, the incidence of bone and liver metastases was synergistically reduced and survival was improved when used in combination with antiprogrammed cell death protein 1 (PD-1) and anti-CTLA-4 ICIs [142]. Therefore, the combination therapy with PRC1-targeted agents and ICIs may be a new therapeutic strategy for DNPC.

Targeting CCL2 signaling, driven downstream of PRC1, is also attractive. Experimental reports have shown that stimulation of androgen-independent PC cells with CCL2 increased proliferation during cabazitaxel treatment, while CCR2 antagonist suppressed the proliferation of cabazitaxel-resistant PC cells during cabazitaxel treatment [143]. Many studies have demonstrated that the CCL2–CCR2 axis plays an important role in the tumor progression of various cancers. However, no clinically approved drugs that can modulate the CCL2–CCR2 axis as anticancer agents for PC exists. Thus, future developments are expected [144].

Precision medicine based on genetic testing may be worth considering for DNPC, for which no breakthrough treatment exists. In particular, a recent study suggested that men with metastatic PC are a population enriched in genetic defects in DNA repair and the overall frequency of germline abnormalities in genes involved in maintaining DNA integrity is 11.8% [145]. Alterations in DNA repair are observed in about 20% of mCRPC, which are the most common mutations in homologous recombination genes (e.g., BRCA1, BRCA2, and ATM) [7]. In a recent open-label phase-III trial, men with mCRPC who had tumors with at least one change in BRCA1, BRCA2, or ATM showed the clinical benefit from olaparib, PARP inhibitor [146].

In 2017, the US Food and Drug Administration approved the use of pembrolizumab, an anti-PD-1 antibody, for patients with solid tumors with microsatellite instability-high (MSI-H) or mismatch repair deficient (dMMR), whose disease has progressed with prior therapy, and for whom no alternative treatment is available. ICIs have been shown the potential for sustained efficacy although the MSI-H/dMMR molecular phenotype has been reported to be rare in PC. Approximately 2% and 3.1% of 1048 and 1033 patients with PC had MSI-H [147] and MSI-H/dMMR status [148], respectively. Thus, the presence of mutations in homologous DNA repair genes (e.g., BRCA1, BRCA2, or ATM) or in MSI-H/dMMR indicates eligibility for PARP inhibitors or ICIs, respectively, and may also be applicable to DNPC treatment. However, the proportion of patients with DNPC who can be treated with such accents is extremely limited.

Bipolar androgen therapy (BAT) refers to an innovative treatment approach for mCRPC that consists of the association of testosterone injections with regular ADT to reach transient supraphysiological testosterone levels [149]. Several clinical trials have reported the therapeutic effect of BAT on PSA decline and assessable imaging improvement [149,150]. BAT efficacy was recently evaluated in an open-label, phase-II, multicohort study of patients with mCRPC whose tumors had progressed after enzalutamide treatment. Consequently, 30% of patients achieved a PSA response. Of those whose tumors progressed after BAT, 52% regained PSA response with enzalutamide treatment [151]. The supraphysiological stimulation of AR by BAT negatively affects metastatic PC cells through various mechanisms (i.e., inhibition of cell cycle progression, induction of direct DNA damage, apoptosis and cancer cell senescence, and reverting CRPC cells to proliferating machinery) that depends on AR activation rather than alternative pathways [149,152]. Interestingly, the BAT clinical trials alone (NCT03522064) or in combination with ICI or PARP inhibitor for CRPC (NCT03516812 or NCT03554317, respectively) are ongoing, the results are awaited, and further applications in DNPC are expected in the future.

## 5. Conclusions

**Figure 3:**
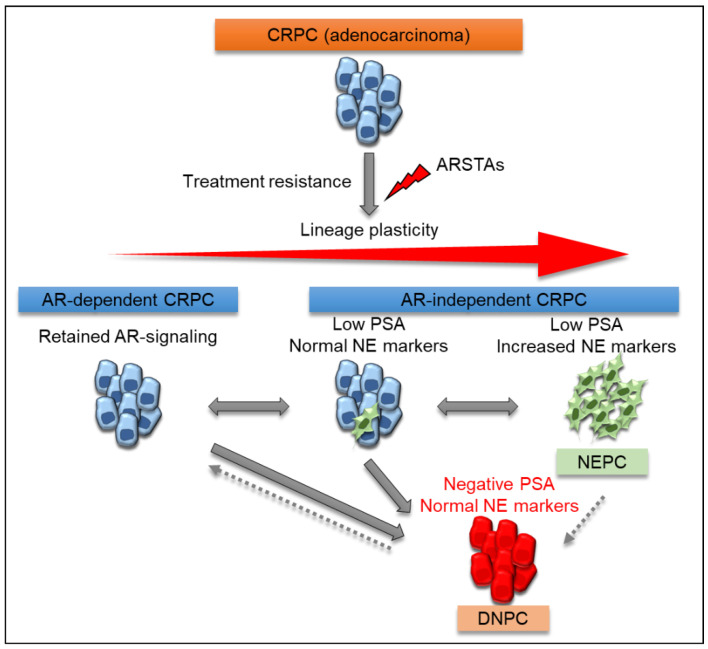
Schematic presentation of the representative transition states the underlying lineage plasticity that occurs during CRPC progression following strong suppression of AR signaling. The long-term use of potent ARSTAs and additional molecular modifications were suggested to may be eventually lead to lethal DNPC via the biological changes between AR-dependent CRPC and NEPC. AR androgen receptor, NE neuroendocrine, ARSTAs AR signaling-targeted agents, CRPC castration-resistant prostate cancer, NEPC neuroendocrine prostate cancer, DNPC double-negative CRPC.

Comparisons of the complex spectrum of phenotypes in three subsets of CRPC (i.e., AR-dependent CRPC, NEPC, and DNPC) are summarized in Table 1. Understanding the dynamics of tumor cell plasticity during the transition from the androgen-responsive to the androgen-unresponsive state may be critical to targeting PC progression (Figure 3). Intensive inhibition of AR signaling with recently improved ARSTAs causes undesirable status in PC cells resulting in an increase of DNPC incidence while such agents actually prolonged survival of PC patient. The biological and pathological phase of PC on which we should focus is shifting from AR-dependent to AR-independent and we should not regard DNPC as a subtype of PC any longer. In DNPC, the mechanism of cancer progression is totally different from AR-dependent CRPC, therefore, different signaling from AR and AR-related signaling as a treatment target must be investigated hereafter. To date, few treatment targets for DNPC have so far been reported as shown in Table 1, and targeting these molecular and epigenetic mechanisms involved in lineage plasticity is expected to improve the diagnosis and treatment of aggressive DNPC.

## Figures and Tables

**Table 1:** Overview of three CRPC subsets; AR-dependent CRPC, NEPC, and DNPC.

	AR-Dependent CRPC	Ref.	NEPC	Ref.	DNPC	Ref.
Prevalence	ARSTA pre-approval era; 88.4%	[1]	ARSTA pre-approval era; 6.3%	[1]	ARSTA pre-approval era; 5.4%	[1]
	ARSTA post-approval era; 63.3%		ARSTA post-approval era; 13.3%		ARSTA post-approval era; 23.3%	
Prognosis	15–32 months	[12]	7–16 months	[11,125,126]	No data reported	
Serum markers	Rising PSA		No—low PSA		No—low PSA	
	Normal range of CgA/NSE/Pro-GRP		Rising CgA/NSE/Pro-GRP		Normal range of CgA/NSE/Pro-GRP	
Histological features	AR positive	[9]	Variable AR level	[9]	Variable AR level	[9]
	Adenocarcinoma		Small cell carcinoma or neuroendocrine differentiation		No neuroendocrine differentiation	
Clinical features	Increasing bone and lymph node metastases in general	[11]	Increasing visceral metastases (e.g., liver and adrenal gland)	[11]	Increasing visceral metastases (e.g., liver and adrenal gland)	[11]
Molecular features	AR mutations	[2,3,4,41,45,51,54,55,56]	EZH2 overexpression	[8,68,69,70,71]	FGF/MAPK pathway	[1,132,140]
	AR amplifications		N-Myc amplification		C-Myc overexpression	
	AR splice variants		AURKA gain		PTEN loss	
	AR bypass/crosstalk pathways; (e.g., growth factors, cytokines, PI3K/AKT, Wnt and GR upregulation)		BRN2, SOX2, PEG10 upregulation		CCL2 upregulation via PRC1 activation	
			RB1, TP53 loss			
			REST downregulation			
Treatments	Taxane anticancer agent; docetaxel and cabazitaxel	[12,118]	Platinum-based chemotherapy; cisplatin or carboplatin/etoposide, cisplatin or carboplatin/docetaxel or irinotecan	[61,118,119,120,121,122,123,124,127]	No effective treatments	[1,142,144,146,147,148]
	ARSTAs; apalutamide, darolutamide, enzalutamide, abiraterone acetate		Immune checkpoint inhibitors; nivolumab, Ipilimumab		Possible of FGF/MAPK blockade	
			AURKA inhibitor (Alisertib)		Possible of PRC1 inhibitor (GW-516)	
			EZH2 inhibitor (PF-06821497, CPI-1205)		Possible targeting CCL2 axis downstream of PRC1	

AR = androgen receptor; NE = neuroendocrine; ARSTA = AR signaling-targeted agent, CRPC = castration resistant prostate cancer; NEPC = neuroendocrine prostate cancer; DNPC = double-negative CRPC; PSA = prostate specific antigen; CgA = chromogranin A; NSE = neuron-specific enolase; Pro-GRP = progastrin-releasing peptide.

## Data Availability

Not applicable.

## References

[1] Bluemn E.G., Coleman I.M., Lucas J.M., Coleman R.T., Hernandez-Lopez S., Tharakan R., Bianchi-Frias D., Dumpit R.F., Kaipainen A., Corella A.N. (2017). Androgen Receptor Pathway-Independent Prostate Cancer Is Sustained through FGF Signaling. Cancer Cell.

[2] Feldman B.J., Feldman D. (2001). The development of androgen-independent prostate cancer. Nat. Rev. Cancer.

[3] Yu Z., Chen S., Sowalsky A.G., Voznesensky O.S., Mostaghel E.A., Nelson P.S., Cai C., Balk S.P. (2014). Rapid Induction of Androgen Receptor Splice Variants by Androgen Deprivation in Prostate Cancer. Clin. Cancer Res..

[4] Grasso C.S., Wu Y.-M., Robinson D.R., Cao X., Dhanasekaran S.M., Khan A.P., Quist M.J., Jing X., Lonigro R.J., Brenner J.C. (2012). The mutational landscape of lethal castration-resistant prostate cancer. Nature.

[5] Parimi V., Goyal R., Poropatich K., Yang X.J. (2014). Neuroendocrine differentiation of prostate cancer: A review. Am. J. Clin. Exp. Urol..

[6] Aggarwal R., Huang J., Alumkal J.J., Zhang L., Feng F.Y., Thomas G.V., Weinstein A.S., Friedl V., Zhang C., Witte O.N. (2018). Clinical and Genomic Characterization of Treatment-Emergent Small-Cell Neuroendocrine Prostate Cancer: A Multi-institutional Prospective Study. J. Clin. Oncol..

[7] Abida W., Cyrta J., Heller G., Prandi D., Armenia J., Coleman I., Cieslik M., Benelli M., Robinson D., Van Allen E.M. (2019). Genomic correlates of clinical outcome in advanced prostate cancer. Proc. Natl. Acad. Sci. USA.

[8] Beltran H., Prandi D., Mosquera J.M., Benelli M., Puca L., Cyrta J., Marotz C., Giannopoulou E., Chakravarthi B.V., Varambally S. (2016). Divergent clonal evolution of castration-resistant neuroendocrine prostate cancer. Nat. Med..

[9] Beltran H., Hruszkewycz A., Scher H.I., Hildesheim J., Isaacs J., Yu E.Y., Kelly K., Lin D., Dicker A.P., Arnold J.T. (2019). The role of lineage plasticity in prostate cancer therapy resistance. Clin. Cancer Res..

[10] Tiwari R., Manzar N., Ateeq B. (2020). Dynamics of Cellular Plasticity in Prostate Cancer Progression. Front. Mol. Biosci..

[11] Iwamoto H., Izumi K., Shimada T., Kano H., Kadomoto S., Makino T., Naito R., Yaegashi H., Shigehara K., Kadono Y. (2021). Androgen receptor signaling-targeted therapy and taxane chemotherapy induce visceral metastasis in castration-resistant prostate cancer. Prostate.

[12] Teo M.Y., Rathkopf D.E., Kantoff P. (2019). Treatment of Advanced Prostate Cancer. Annu. Rev. Med..

[13] Waltering K.K., Urbanucci A., Visakorpi T. (2012). Androgen receptor (AR) aberrations in castration-resistant prostate cancer. Mol. Cell. Endocrinol..

[14] Gottlieb B., Beitel L.K., Nadarajah A., Paliouras M., Trifiro M. (2012). The Androgen Receptor Gene Mutations Database The androgen receptor gene mutations database: 2012 Update. Hum. Mutat..

[15] Nadiminty N., Gao A.C. (2011). Mechanisms of persistent activation of the androgen receptor in CRPC: Recent advances and future perspectives. World J. Urol..

[16] Heinlein C.A., Chang C. (2004). Androgen receptor in prostate cancer. Endocr. Rev..

[17] Steketee K., Timmerman L., Der Made A.C.Z.-V., Doesburg P., Brinkmann A.O., Trapman J. (2002). Broadened ligand responsiveness of androgen receptor mutants obtained by random amino acid substitution of H874 and mutation hot spot T877 in prostate cancer. Int. J. Cancer.

[18] Robinson D., Van Allen E.M., Wu Y.-M., Schultz N., Lonigro R.J., Mosquera J.-M., Montgomery B., Taplin M.-E., Pritchard C.C., Attard G. (2015). Integrative Clinical Genomics of Advanced Prostate Cancer. Cell.

[19] Balbas M.D., Evans M.J., Hosfield D.J., Wongvipat J., Arora V.K., A Watson P., Chen Y., Greene G.L., Shen Y., Sawyers C.L. (2013). Overcoming mutation-based resistance to antiandrogens with rational drug design. eLife.

[20] Obinata D., Lawrence M.G., Takayama K., Choo N., Risbridger G.P., Takahashi S., Inoue S. (2020). Recent Discoveries in the Androgen Receptor Pathway in Castration-Resistant Prostate Cancer. Front. Oncol..

[21] Linja M.J., Savinainen K.J., Saramäki O.R., Tammela T.L., Vessella R.L., Visakorpi T. (2001). Amplification and overexpression of androgen receptor gene in hormone-refractory prostate cancer. Cancer Res..

[22] Koivisto P.A., Helin H.J. (1999). Androgen receptor gene amplification increases tissue PSA protein expression in hormone-refractory prostate carcinoma. J. Pathol..

[23] Grossmann M.E., Huang H., Tindall N.J. (2001). Androgen receptor signaling in androgen-refractory prostate cancer. J. Natl. Cancer Inst..

[24] Gregory C.W., Johnson R.T., Mohler J.L., French F.S., Wilson E.M. (2001). Androgen receptor stabilization in recurrent prostate cancer is associated with hypersensitivity to low androgen. Cancer Res..

[25] Quigley D.A., Dang H.X., Zhao S.G., Lloyd P., Aggarwal R., Alumkal J.J., Foye A., Kothari V., Perry M.D., Bailey A.M. (2018). Genomic Hallmarks and Structural Variation in Metastatic Prostate Cancer. Cell.

[26] Takeda D.Y., Spisák S., Seo J.-H., Bell C., O’Connor E., Korthauer K., Ribli D., Csabai I., Solymosi N., Szállási Z. (2018). A Somatically Acquired Enhancer of the Androgen Receptor Is a Noncoding Driver in Advanced Prostate Cancer. Cell.

[27] Viswanathan S.R., Ha G., Hoff A.M., Wala J.A., Carrot-Zhang J., Whelan C.W., Haradhvala N.J., Freeman S.S., Reed S.C., Rhoades J. (2018). Structural Alterations Driving Castration-Resistant Prostate Cancer Revealed by Linked-Read Genome Sequencing. Cell.

[28] Zhang L., Altuwaijri S., Deng F., Chen L., Lal P., Bhanot U.K., Korets R., Wenske S., Lilja H.G., Chang C. (2009). NF-kappaB regulates androgen receptor expression and prostate cancer growth. Am. J. Pathol..

[29] Dehm S.M., Tindall D.J. (2011). Alternatively spliced androgen receptor variants. Endocr. Relat. Cancer.

[30] Guo Z., Yang X., Sun F., Jiang R., Linn D.E., Chen H., Chen H., Kong X., Melamed J., Tepper C.G. (2009). A Novel Androgen Receptor Splice Variant Is Up-regulated during Prostate Cancer Progression and Promotes Androgen Depletion–Resistant Growth. Cancer Res..

[31] Sun S., Sprenger C.C., Vessella R.L., Haugk K., Soriano K., Mostaghel E.A., Page S.T., Coleman I.M., Nguyen H.M., Sun H. (2010). Castration resistance in human prostate cancer is conferred by a frequently occurring androgen receptor splice variant. J. Clin. Investig..

[32] Hu R., Dunn T.A., Wei S., Isharwal S., Veltri R.W., Humphreys E., Han M., Partin A.W., Vessella R.L., Isaacs W.B. (2009). Ligand-Independent Androgen Receptor Variants Derived from Splicing of Cryptic Exons Signify Hormone-Refractory Prostate Cancer. Cancer Res..

[33] Nakazawa M., Lu C., Chen Y., Paller C.J., Carducci M.A., Eisenberger M.A., Luo J., Antonarakis E.S. (2015). Serial blood-based analysis of AR-V7 in men with advanced prostate cancer. Ann. Oncol..

[34] Sharp A., Coleman I., Yuan W., Sprenger C., Dolling D., Rodrigues D.N., Russo J.W., Figueiredo I., Bertan C., Seed G. (2018). Androgen receptor splice variant-7 expression emerges with castration resistance in prostate cancer. J. Clin. Investig..

[35] Liu X., Ledet E., Li D., Dotiwala A., Steinberger A., Feibus A., Li J., Qi Y., Silberstein J., Lee B. (2016). A Whole Blood Assay for AR-V7 and AR v567es in Patients with Prostate Cancer. J. Urol..

[36] Liu G., Sprenger C., Sun S., Epilepsia K.S., Haugk K., Zhang X., Coleman I., Nelson P.S., Plymate S. (2013). AR Variant ARv567es Induces Carcinogenesis in a Novel Transgenic Mouse Model of Prostate Cancer. Neoplasia.

[37] Hörnberg E., Ylitalo E.B., Crnalic S., Antti H., Stattin P., Widmark A., Bergh A., Wikström P. (2011). Expression of Androgen Receptor Splice Variants in Prostate Cancer Bone Metastases is Associated with Castration-Resistance and Short Survival. PLoS ONE.

[38] Watson P.A., Chen Y.F., Balbas M.D., Wongvipat J., Socci N.D., Viale A., Kim K., Sawyers C.L. (2010). Constitutively active androgen receptor splice variants expressed in castration-resistant prostate cancer require full-length androgen receptor. Proc. Natl. Acad. Sci. USA.

[39] Zhang T., Karsh L.I., Nissenblatt M.J., Canfield S.E. (2020). Androgen Receptor Splice Variant, AR-V7, as a Biomarker of Resistance to Androgen Axis-Targeted Therapies in Advanced Prostate Cancer. Clin. Genitourin. Cancer.

[40] Messner E.A., Steele T.M., Tsamouri M.M., Hejazi N., Gao A.C., Mudryj M., Ghosh P.M. (2020). The Androgen Receptor in Prostate Cancer: Effect of Structure, Ligands and Spliced Variants on Therapy. Biomedicines.

[41] Culig Z., Hobisch A., Cronauer M.V., Radmayr C., Trapman J., Hittmair A., Bartsch G., Klocker H. (1994). Androgen receptor activation in prostatic tumor cell lines by insulin-like growth factor-I, keratinocyte growth factor, and epidermal growth factor. Cancer Res..

[42] Wu J.D., Haugk K., Woodke L., Nelson P., Coleman I., Plymate S.R. (2006). Interaction of IGF signaling and the androgen receptor in prostate cancer progression. J. Cell. Biochem..

[43] Hua Y., Camarco D.P., Strock C.J., Johnston P.A., Trask O.J. (2017). High Content Positional Biosensor Assay to Screen for Compounds that Prevent or Disrupt Androgen Receptor and Transcription Intermediary Factor 2 Protein-Protein Interactions. Adv. Struct. Saf. Stud..

[44] Saraon P., Jarvi K., Diamandis E.P. (2011). Molecular Alterations during Progression of Prostate Cancer to Androgen Independence. Clin. Chem..

[45] Medzhitov R. (2010). Inflammation 2010: New Adventures of an Old Flame. Cell.

[46] Malinowska K., Neuwirt H., Cavarretta I.T., Bektic J., Steiner H., Dietrich H., Moser P.L., Fuchs D., Hobisch A., Culig Z. (2009). Interleukin-6 stimulation of growth of prostate cancer in vitro and in vivo through activation of the androgen receptor. Endocr. Relat. Cancer.

[47] Nguyen D.P., Li J., Tewari A.K. (2014). Inflammation and prostate cancer: The role of interleukin 6 (IL-6). BJU Int..

[48] Waugh D.J., Wilson C. (2008). The Interleukin-8 Pathway in Cancer. Clin. Cancer Res..

[49] Toren P., Zoubeidi A. (2014). Targeting the PI3K/Akt pathway in prostate cancer: Challenges and opportunities (Review). Int. J. Oncol..

[50] Fruman D.A., Rommel C. (2014). PI3K and cancer: Lessons, challenges and opportunities. Nat. Rev. Drug Discov..

[51] Carver B.S., Chapinski C., Wongvipat J., Hieronymus H., Chen Y., Chandarlapaty S., Arora V.K., Le C., Koutcher J., Scher H. (2011). Reciprocal Feedback Regulation of PI3K and Androgen Receptor Signaling in PTEN-Deficient Prostate Cancer. Cancer Cell.

[52] Chandarlapaty S. (2012). Negative Feedback and Adaptive Resistance to the Targeted Therapy of Cancer. Cancer Discov..

[53] Thomas C., Lamoureux F., Crafter C., Davies B.R., Beraldi E., Fazli L., Kim S., Thaper D., Gleave M.E., Zoubeidi A. (2013). Synergistic Targeting of PI3K/AKT Pathway and Androgen Receptor Axis Significantly Delays Castration-Resistant Prostate Cancer Progression In Vivo. Mol. Cancer Ther..

[54] Truica C.I., Byers S., Gelmann E.P. (2000). Beta-catenin affects androgen receptor transcriptional activity and ligand specificity. Cancer Res..

[55] Zhang Z., Cheng L., Li J., Farah E., Atallah N.M., Pascuzzi P.E., Gupta S., Liu X. (2018). Inhibition of the Wnt/beta-catenin pathway overcomes resistance to enzalutamide in castration-resistant prostate cancer. Cancer Res..

[56] Buttigliero C., Tucci M., Bertaglia V., Vignani F., Bironzo P., Di Maio M., Scagliotti G.V. (2015). Understanding and overcoming the mechanisms of primary and acquired resistance to abiraterone and enzalutamide in castration resistant prostate cancer. Cancer Treat. Rev..

[57] Isikbay M., Otto K., Kregel S., Kach J., Cai Y., Griend N.J.V., Conzen S.D., Szmulewitz R.Z. (2014). Glucocorticoid receptor activity contributes to resistance to androgen-targeted therapy in prostate cancer. Horm. Cancer.

[58] Arora V.K., Schenkein E., Murali R., Subudhi S.K., Wongvipat J., Balbas M.D., Shah N., Cai L., Efstathiou E., Logothetis C. (2013). Glucocorticoid Receptor Confers Resistance to Antiandrogens by Bypassing Androgen Receptor Blockade. Cell.

[59] Puhr M., Hoefer J., Eigentler A., Ploner C., Handle F., Schaefer G., Kroon J., Leo A., Heidegger I.M., Eder I. (2017). The Glucocorticoid Receptor Is a Key Player for Prostate Cancer Cell Survival and a Target for Improved Antiandrogen Therapy. Clin. Cancer Res..

[60] Epstein J.I., Amin M.B., Beltran H., Lotan T.L., Mosquera J.-M., Reuter V.E., Robinson B.D., Troncoso P., Rubin M.A. (2014). Proposed Morphologic Classification of Prostate Cancer with Neuroendocrine Differentiation. Am. J. Surg. Pathol..

[61] Aparicio A.M., Harzstark A.L., Corn P.G., Wen S., Araujo J.C., Tu S.-M., Pagliaro L.C., Kim J., Millikan R.E., Ryan C.J. (2013). Platinum-Based Chemotherapy for Variant Castrate-Resistant Prostate Cancer. Clin. Cancer Res..

[62] Huang J., Yao J.L., di Sant’Agnese P.A., Yang Q., Bourne P.A., Na Y. (2006). Immunohistochemical characterization of neuroendocrine cells in prostate cancer. Prostate.

[63] Nakada S.Y., di Sant’Agnese P.A., Moynes R.A., Hiipakka R.A., Liao S., Cockett A.T., Abrahamsson P.A. (1993). The androgen receptor status of neuroendocrine cells in human benign and malignant prostatic tissue. Cancer Res..

[64] Ellis L., Loda M. (2018). LSD1: A single target to combat lineage plasticity in lethal prostate cancer. Proc. Natl. Acad. Sci. USA.

[65] Smith B.A., Sokolov A., Uzunangelov V., Baertsch R., Newton Y., Graim K., Mathis C., Cheng D., Stuart J.M., Witte O.N. (2015). A basal stem cell signature identifies aggressive prostate cancer phenotypes. Proc. Natl. Acad. Sci. USA.

[66] Fletcher C.E. (2019). The Role of Splicing Regulators in the Emergence of Treatment-induced Neuroendocrine Prostate Cancer: The Next Generation of Drug Targets?. Eur. Urol..

[67] Davies A., Zoubeidi A., A Selth L. (2020). The epigenetic and transcriptional landscape of neuroendocrine prostate cancer. Endocr. Relat. Cancer.

[68] Davies A.H., Beltran H., Zoubeidi A. (2018). Cellular plasticity and the neuroendocrine phenotype in prostate cancer. Nat. Rev. Urol..

[69] Beltran H., Rickman D.S., Park K., Chae S.S., Sboner A., Macdonald T.Y., Wang Y., Sheikh K.L., Terry S., Tagawa S.T. (2011). Molecular Characterization of Neuroendocrine Prostate Cancer and Identification of New Drug Targets. Cancer Discov..

[70] Colleen M., Phillips J.W., Smith B.A., Park J.W., Stoyanova T., McCaffrey E.F., Baertsch R., Sokolov A., Meyerowitz J.G., Mathis C. (2016). N-Myc Drives Neuroendocrine Prostate Cancer Initiated from Human Prostate Epithelial Cells. Cancer Cell.

[71] Dardenne E., Beltran H., Benelli M., Gayvert K., Berger A., Puca L., Cyrta J., Sboner A., Noorzad Z., Macdonald T. (2016). N-Myc Induces an EZH2-Mediated Transcriptional Program Driving Neuroendocrine Prostate Cancer. Cancer Cell.

[72] Yamada Y., Beltran H. (2021). Clinical and Biological Features of Neuroendocrine Prostate Cancer. Curr. Oncol. Rep..

[73] Mu P., Zhang Z., Benelli M., Karthaus W.R., Hoover E., Chen C.-C., Wongvipat J., Ku S.-Y., Gao D., Cao Z. (2017). SOX2 promotes lineage plasticity and antiandrogen resistance in TP53-and RB1-deficient prostate cancer. Science.

[74] Ku S.Y., Rosario S., Wang Y., Mu P., Seshadri M., Goodrich Z.W., Goodrich M.M., Labbé D.P., Gomez E.C., Wang J. (2017). Rb1 and Trp53 cooperate to suppress prostate cancer lineage plasticity, metastasis, and antiandrogen resistance. Science.

[75] Li Y., Donmez N., Sahinalp C., Xie N., Wang Y., Xue H., Mo F., Beltran H., Gleave M., Wang Y. (2017). SRRM4 Drives Neuroendocrine Transdifferentiation of Prostate Adenocarcinoma Under Androgen Receptor Pathway Inhibition. Eur. Urol..

[76] Zhang X., Coleman I.M., Brown L.G., True L.D., Kollath L., Lucas J.M., Lam H.-M., Dumpit R., Corey E., Chéry L. (2015). SRRM4 Expression and the Loss of REST Activity May Promote the Emergence of the Neuroendocrine Phenotype in Castration-Resistant Prostate Cancer. Clin. Cancer Res..

[77] Bishop J.L., Thaper D., Vahid S., Davies A., Ketola K., Kuruma H., Jama R., Nip K.M., Angeles A., Johnson F. (2017). The Master Neural Transcription Factor BRN2 Is an Androgen Receptor–Suppressed Driver of Neuroendocrine Differentiation in Prostate Cancer. Cancer Discov..

[78] Yu X., Cates J.M., Morrissey C., You C., Grabowska M.M., Zhang J., DeGraff D.J., Strand D.W., Franco O.E., Lin-Tsai O. (2014). SOX2 expression in the developing, adult, as well as, diseased prostate. Prostate Cancer Prostatic Dis..

[79] Kareta M.S., Gorges L.L., Hafeez S., Benayoun B.A., Marro S., Zmoos A.-F., Cecchini M.J., Spacek D., Batista L.F., O’Brien M. (2015). Inhibition of Pluripotency Networks by the Rb Tumor Suppressor Restricts Reprogramming and Tumorigenesis. Cell Stem Cell.

[80] Choi Y.J., Lin C.-P., Ho J.J., He X., Okada N., Bu P., Zhong Y., Kim S.Y., Bennett M.J., Chen C. (2011). miR-34 miRNAs provide a barrier for somatic cell reprogramming. Nat. Cell Biol..

[81] Akamatsu S., Wyatt A.W., Lin D., Lysakowski S., Zhang F., Kim S., Tse C., Wang K., Mo F., Haegert A. (2015). The Placental Gene PEG10 Promotes Progression of Neuroendocrine Prostate Cancer. Cell Rep..

[82] Wang C., Xiao Y., Hu Z., Chen Y., Liu N., Hu G. (2008). PEG10 directly regulated by E2Fs might have a role in the development of hepatocellular carcinoma. FEBS Lett..

[83] Kim S., Thaper D., Bidnur S., Toren P., Akamatsu S., Bishop J.L., Colins C., Vahid S., Zoubeidi A. (2019). PEG10 is associated with treatment-induced neuroendocrine prostate cancer. J. Mol. Endocrinol..

[84] Dominguez-Brauer C., Thu K.L., Mason J.M., Blaser H., Bray M.R., Mak T.W. (2015). Targeting Mitosis in Cancer: Emerging Strategies. Mol. Cell.

[85] Mosquera J.M., Beltran H., Park K., Macdonald T.Y., Robinson B.D., Tagawa S.T., Perner S., Bismar T.A., Erbersdobler A., Dhir R. (2013). Concurrent AURKA and MYCN Gene Amplifications Are Harbingers of Lethal TreatmentRelated Neuroendocrine Prostate Cancer. Neoplasia.

[86] Zou M., Toivanen R., Mitrofanova A., Floch N., Hayati S., Sun Y., Le Magnen C., Chester D., Mostaghel E.A., Califano A. (2017). Transdifferentiation as a Mechanism of Treatment Resistance in a Mouse Model of Castration-Resistant Prostate Cancer. Cancer Discov..

[87] Saxby H., Mikropoulos C., Boussios S. (2020). An Update on the Prognostic and Predictive Serum Biomarkers in Metastatic Prostate Cancer. Diagnostics.

[88] Sargos P., Ferretti L., Gross-Goupil M., Orre M., Cornelis F., De Figueiredo B.H., Houédé N., Merino C., Roubaud G., Dallaudiére B. (2014). Characterization of prostate neuroendocrine cancers and therapeutic management: A literature review. Prostate Cancer Prostatic Dis..

[89] Kamiya N., Akakura K., Suzuki H., Isshiki S., Komiya A., Ueda T., Ito H. (2003). Pretreatment serum level of neuron specific enolase (NSE) as a prognostic factor in metastatic prostate cancer patients treated with endocrine therapy. Eur. Urol..

[90] Heck M.M., Thaler M.A., Schmid S.C., Seitz A.-K., Tauber R., Kübler H., Maurer T., Thalgott M., Hatzichristodoulou G., Höppner M. (2017). Chromogranin A and neurone-specific enolase serum levels as predictors of treatment outcome in patients with metastatic castration-resistant prostate cancer undergoing abiraterone therapy. BJU Int..

[91] Molina R., Filella X., Augé J.M. (2004). ProGRP: A new biomarker for small cell lung cancer. Clin. Biochem..

[92] Korse C.M., Taal B.G., Vincent A., van Velthuysen M.-L.F., Baas P., Buning-Kager J.C., Linders T.C., Bonfrer J.M. (2012). Choice of tumour markers in patients with neuroendocrine tumours is dependent on the histological grade. A marker study of Chromogranin A, Neuron specific enolase, Progastrin-releasing peptide and cytokeratin fragments. Eur. J. Cancer.

[93] Korse C.M., Taal B.G., Bonfrer J.M.G., Vincent A., Van Velthuysen M.L., Baas P. (2011). An elevated progastrin-releasing peptide level in patients with well-differentiated neuroendocrine tumours indicates a primary tumour in the lung and predicts a shorter survival. Ann. Oncol..

[94] Martucci F., Pascale M., Valli M.C., Pesce G.A., Froesch P., Giovanella L., Richetti A., Treglia G. (2020). Impact of 18F-FDG PET/CT in Staging Patients With Small Cell Lung Cancer: A Systematic Review and Meta-Analysis. Front. Med..

[95] Spratt D.E., Gavane S., Tarlinton L., Fareedy S.B., Doran M.G., Zelefsky M.J., Osborne J.R. (2014). Utility of FDG-PET in clinical neuroendocrine prostate cancer. Prostate.

[96] Lamberts S., Van Der Lely A., Hofland L. (2002). New somatostatin analogs: Will they fulfil old promises?. Eur. J. Endocrinol..

[97] Patel Y.C. (1999). Somatostatin and Its Receptor Family. Front. Neuroendocr..

[98] Rai U., Thrimawithana T.R., Valery C., Young S.A. (2015). Therapeutic uses of somatostatin and its analogues: Current view and potential applications. Pharmacol. Ther..

[99] Grozinsky-Glasberg S., Shimon I., Korbonits M.A., Grossman A.B. (2008). Somatostatin analogues in the control of neuroendocrine tumours: Efficacy and mechanisms. Endocr. Relat. Cancer.

[100] Spieth M.E., Lin Y.G., Nguyen T.T. (2002). Diagnosing and Treating Small-Cell Carcinomas of Prostatic Origin. Clin. Nucl. Med..

[101] Mori H., Nakajima K., Kadomoto S., Mizokami A., Ikeda H., Wakabayashi H., Kinuya S. (2018). Imaging Somatostatin Receptor Activity in Neuroendocrine-differentiated Prostate Cancer. Intern. Med..

[102] Usmani S., Orevi M., Stefanelli A., Zaniboni A., Gofrit O.N., Bnà C., Illuminati S., Lojacono G., Noventa S., Savelli G. (2019). Neuroendocrine differentiation in castration resistant prostate cancer. Nuclear medicine radiopharmaceuticals and imaging techniques: A narrative review. Crit. Rev. Oncol..

[103] Gomes-Porras M., Cárdenas-Salas J., Álvarez-Escolá C. (2020). Somatostatin Analogs in Clinical Practice: A Review. Int. J. Mol. Sci..

[104] Binderup T., Knigge U., Loft A., Mortensen J., Pfeifer A., Federspiel B., Hansen C.P., Højgaard L., Kjaer A. (2010). Functional Imaging of Neuroendocrine Tumors: A Head-to-Head Comparison of Somatostatin Receptor Scintigraphy, 123I-MIBG Scintigraphy, and 18F-FDG PET. J. Nucl. Med..

[105] Garin E., Le Jeune F., Devillers A., Cuggia M., De Lajarte-Thirouard A.-S., Bouriel C., Boucher E., Raoul J.-L. (2009). Predictive Value of 18F-FDG PET and Somatostatin Receptor Scintigraphy in Patients with Metastatic Endocrine Tumors. J. Nucl. Med..

[106] Sainio M., Visakorpi T., Tolonen T., Ilvesaro J., Bova G.S. (2018). Expression of neuroendocrine differentiation markers in lethal metastatic castration-resistant prostate cancer. Pathol. Res. Pract..

[107] Aggarwal R., Zhang T., Small E.J., Armstrong A.J. (2014). Neuroendocrine Prostate Cancer: Subtypes, Biology, and Clinical Outcomes. J. Natl. Compr. Cancer Netw..

[108] Winkler H., Westhead E. (1980). Th molecular organization of adrenal chromaffin granules. Neuroscience.

[109] Nilsson O., Jakobsen A.-M.L., Kölby L., Bernhardt P., Forssell-Aronsson E., Ahlman H. (2004). Importance of Vesicle Proteins in the Diagnosis and Treatment of Neuroendocrine Tumors. Ann. N. Y. Acad. Sci..

[110] Portela-Gomes G.M., Lukinius A., Grimelius L. (2000). Synaptic Vesicle Protein 2, A New Neuroendocrine Cell Marker. Am. J. Pathol..

[111] Rindi G., Paolotti D., Fiocca R., Wiedenmann B., Henry J.-P., Solcia E. (2000). Vesicular monoamine transporter 2 as a marker of gastric enterochromaffin-like cell tumors. Virchows Arch..

[112] Söllner T., Bennett M.K., Whiteheart S.W., Scheller R.H., Rothman J.E. (1993). A protein assembly-disassembly pathway in vitro that may correspond to sequential steps of synaptic vesicle docking, activation, and fusion. Cell.

[113] Tahara S., Sanno N., Teramoto A., Osamura R.Y. (1999). Expression of Rab3, a Ras-related GTP-binding protein, in human non-tumorous pituitaries and pituitary adenomas. Mod. Pathol..

[114] Regazzi R., Wollheim C., Lang J., Theler J., Rossetto O., Montecucco C., Sadoul K., Weller U., Palmer M., Thorens B. (1995). VAMP-2 and cellubrevin are expressed in pancreatic beta-cells and are essential for Ca(2+)-but not for GTP gamma S-induced insulin secretion. EMBO J..

[115] Braun J.E., Fritz B.A., Wong S.M., Lowe A.W. (1994). Identification of a vesicle-associated membrane protein (VAMP)-like mem-brane protein in zymogen granules of the rat exocrine pancreas. J. Biol. Chem..

[116] Tischler A.S., Mobtaker H., Mann K., Nunnemacher G., Jason W.J., Dayal Y., Delellis R.A., Adelman L., Wolfe H.J. (1986). An-ti-lymphocyte antibody Leu-7 (HNK-1) recognizes a constituent of neuroendocrine granule matrix. J. Histochem. Cytochem..

[117] Lipinski M., Braham K., Caillaud J.M., Carlu C., Tursz T. (1983). HNK-1 antibody detects an antigen expressed on neuroectodermal cells. J. Exp. Med..

[118] Mohler J.L., Antonarakis E.S., Armstrong A.J., D’Amico A.V., Davis B.J., Dorff T., Eastham J.A., Enke C.A., Farrington T.A., Higano C.S. (2019). Prostate Cancer, Version 2.2019, NCCN Clinical Practice Guidelines in Oncology. J. Natl. Compr. Cancer Netw..

[119] Fléchon A., Pouessel D., Ferlay C., Perol D., Beuzeboc P., Gravis G., Joly F., Oudard S., Deplanque G., Zanetta S. (2011). Phase II study of carboplatin and etoposide in patients with anaplastic progressive metastatic castration-resistant prostate Cancer (mCRPC) with or without neuroendocrine differentiation: Results of the French Geni-to-Urinary Tumor Group (GETUG) P01. Trial. Ann. Oncol..

[120] Yamada T., Ohtsubo K., Mouri H., Yamashita K., Yasumoto K., Izumi K., Zen Y., Watanabe H., Yano S. (2009). Combined chemotherapy with carboplatin plus irinotecan showed favorable efficacy in a patient with relapsed small cell carcinoma of the prostate complicated with meningeal carcinomatosis. Int. J. Clin. Oncol..

[121] Aoki H., Ishidoya S., Ito A., Endoh M., Shimazui T., Arai Y. (2006). Experience of the treatment with gemcitabine, docetaxel, and carboplatin (GDC) chemotherapy for patients with small-cell carcinoma of the prostate. Int. J. Urol..

[122] Papandreou C.N., Daliani D.D., Thall P.F., Tu S.-M., Wang X., Reyes A., Troncoso P., Logothetis C.J. (2002). Results of a Phase II Study with Doxorubicin, Etoposide, and Cisplatin in Patients with Fully Characterized Small-Cell Carcinoma of the Prostate. J. Clin. Oncol..

[123] Maesaka F., Nakai Y., Tomizawa M., Owari T., Miyake M., Inoue T., Anai S., Tanaka N., Fujimoto K. (2019). Amrubicin is ef-fective against small cell carcinoma of the prostate as a second-line chemotherapeutic agent: A case report. IJU Case Rep..

[124] Apostolidis L., Nientiedt C., Winkler E.C., Berger A.K., Kratochwil C., Kaiser A., Becker A.-S., Jäger D., Hohenfellner M., Hüttenbrink C. (2019). Clinical characteristics, treatment outcomes and potential novel therapeutic options for patients with neuroendocrine carcinoma of the prostate. Oncotarget.

[125] Wang H.T., Yao Y.H., Li B.G., Tang Y., Chang J.W., Zhang J. (2014). Neuroendocrine prostate Cancer (NEPC) progressing from conventional prostatic adenocarcinoma: Factors associated with time to development of NEPC and survival from NEPC di-agnosis-a systematic review and pooled analysis. J. Clin. Oncol..

[126] Spetsieris N., Boukovala M., Patsakis G., Alafis I., Efstathiou E. (2020). Neuroendocrine and Aggressive-Variant Prostate Cancer. Cancers.

[127] Beltran H., Oromendia C., Danila D.C., Montgomery B., Hoimes C., Szmulewitz R.Z., Vaishampayan U., Armstrong A.J., Stein M., Pinski J. (2019). A phase II trial of the Aurora kinase a inhibitor alisertib for patients with castration-resistant and neuroendocrine prostate cancer: Efficacy and bi-omarkers. Clin. Cancer Res..

[128] Ge R., Wang Z., Montironi R., Jiang Z., Cheng M., Santoni M., Huang K., Massari F., Lu X., Cimadamore A. (2020). Epigenetic modulations and lineage plasticity in advanced prostate cancer. Ann. Oncol..

[129] Hsu E.-C., Rice M.A., Bermudez A., Marques F.J.G., Aslan M., Liu S., Ghoochani A., Zhang C.A., Chen Y.-S., Zlitni A. (2020). Trop2 is a driver of metastatic prostate cancer with neuroendocrine phenotype via PARP. Proc. Natl. Acad. Sci. USA.

[130] Wang W., Epstein J.I. (2008). Small cell carcinoma of the prostate. A morphologic and immunohistochemical study of 95 cases. Am. J Surg. Pathol..

[131] Vellky J.E., Ricke W.A. (2020). Development and prevalence of castration-resistant prostate cancer subtypes. Neoplasia.

[132] Su W., Han H.H., Wang Y., Zhang B., Zhou B., Cheng Y., Rumandla A., Gurrapu S., Chakraborty G., Su J. (2019). The Polycomb repressor Complex 1 drives dou-ble-negative prostate cancer metastasis by coordinating stemness and immune suppression. Cancer Cell..

[133] Izumi K., Mizokami A. (2019). Suppressive Role of Androgen/Androgen Receptor Signaling via Chemokines on Prostate Cancer Cells. J. Clin. Med..

[134] Izumi K., Fang L.Y., Mizokami A., Namiki M., Li L., Lin W.J., Chang C. (2013). Targeting the androgen receptor with siRNA promotes prostate cancer metastasis through enhanced macrophage recruitment via CCL2/CCR2-induced STAT3 activation. EMBO Mol. Med..

[135] Sun Y., Wang B.E., Leong K.G., Yue P., Li L., Jhunjhunwala S., Chen D., Seo K., Modrusan Z., Gao W.Q. (2012). Androgen deprivation causes epithelial-mesenchymal transition in the prostate: Implications for andro-gen-deprivation therapy. Cancer Res..

[136] Viré E., Brenner C., Deplus R., Blanchon L., Fraga M., Didelot C., Morey L., Van Eynde A., Bernard D., Vanderwinden J.-M. (2006). The Polycomb group protein EZH2 directly controls DNA methylation. Nat. Cell Biol..

[137] Acevedo V.D., Gangula R.D., Freeman K.W., Li R., Zhang Y., Wang F., Ayala G.E., Peterson L.E., Ittmann M., Spencer D.M. (2007). Inducible FGFR-1 Activation Leads to Irreversible Prostate Adenocarcinoma and an Epithelial-to-Mesenchymal Transition. Cancer Cell.

[138] Mulholland D.J., Kobayashi N., Ruscetti M., Zhi A., Tran L.M., Huang J., Gleave M., Wu H. (2012). Pten Loss and RAS/MAPK Activation Cooperate to Promote EMT and Metastasis Initiated from Prostate Cancer Stem/Progenitor Cells. Cancer Res..

[139] Oka H., Chatani Y., Kohno M., Kawakita M., Ogawa O. (2005). Constitutive activation of the 41- and 43-kDa mitogen-activated protein (MAP) kinases in the progression of prostate cancer to an androgen-independent state. Int. J. Urol..

[140] Hubbard G.K., Mutton L.N., Khalili M., McMullin R.P., Hicks J.L., Bianchi-Frias D., Horn L.A., Kulac I., Moubarek M.S., Nelson P.S. (2015). Combined MYC Activation and Pten Loss Are Sufficient to Create Genomic Instability and Lethal Metastatic Prostate Cancer. Cancer Res..

[141] Sehrawat A., Gao L., Wang Y., Bankhead A., McWeeney S.K., King C.J., Schwartzman J., Urrutia J., Bisson W.H., Coleman D.J. (2018). LSD1 activates a lethal prostate cancer gene network independently of its demethylase function. Proc. Natl. Acad. Sci. USA.

[142] Shen M.M. (2019). A Positive Step toward Understanding Double-Negative Metastatic Prostate Cancer. Cancer Cell.

[143] Natsagdorj A., Izumi K., Hiratsuka K., Machioka K., Iwamoto H., Naito R., Makino T., Kadomoto S., Shigehara K., Kadono Y. (2018). CCL2 induces resistance to the antiproliferative effect of cabazitaxel in prostate cancer cells. Cancer Sci..

[144] Iwamoto H., Izumi K., Mizokami A. (2020). Is the C-C Motif Ligand 2–C-C Chemokine Receptor 2 Axis a Promising Target for Cancer Therapy and Diagnosis?. Int. J. Mol. Sci..

[145] Pritchard C.C., Mateo J., Walsh M.F., De Sarkar N., Abida W., Beltran H., Garofalo A., Gulati R., Carreira S., Eeles R. (2016). Inherited DNA-Repair Gene Mutations in Men with Metastatic Prostate Cancer. N. Engl. J. Med..

[146] Hussain M., Mateo J., Fizazi K., Saad F., Shore N., Sandhu S., Chi K.N., Sartor O., Agarwal N., Olmos D. (2020). Survival with Olaparib in Metastatic Castration-Resistant Prostate Cancer. N. Engl. J. Med..

[147] Latham A., Srinivasan P., Kemel Y., Shia J., Bandlamudi C., Mandelker D., Middha S., Hechtman J., Zehir A., Dubard-Gault M. (2019). Microsatellite Instability Is Associated with the Presence of Lynch Syndrome Pan-Cancer. J. Clin. Oncol..

[148] Abida W., Cheng M.L., Armenia J., Middha S., Autio K.A., Vargas H.A., Rathkopf D., Morris M.J., Danila D.C., Slovin S.F. (2019). Analysis of the Prevalence of Microsatellite Instability in Prostate Cancer and Response to Immune Checkpoint Blockade. JAMA Oncol..

[149] Leone G., Buttigliero C., Pisano C., Di Stefano R.F., Tabbò F., Turco F., Vignani F., Scagliotti G.V., Di Maio M., Tucci M. (2020). Bipolar androgen therapy in prostate cancer: Current evidences and future perspectives. Crit. Rev. Oncol..

[150] Xie T., Song X.-L., Wang C., Yu Y.-Z., Wang J.-Q., Chen Z.-S., Zhao S.-C. (2021). The role of androgen therapy in prostate cancer: From testosterone replacement therapy to bipolar androgen therapy. Drug Discov. Today.

[151] Teply B.A., Wang H., Luber B., Sullivan R., Rifkind I., Bruns A., Spitz A., DeCarli M., Sinibaldi V., Pratz C.F. (2018). Bipolar androgen therapy in men with metastatic castration-resistant prostate cancer after progression on enzalutamide: An open-label, phase 2, multicohort study. Lancet Oncol..

[152] Mirochnik Y., Veliceasa D., Williams L., Maxwell K., Yemelyanov A., Budunova I., Volpert O.V. (2012). Androgen Receptor Drives Cellular Senescence. PLoS ONE.

